# Design and Characterization of Gelatin-Based Interpenetrating Polymer Networks for Biomedical Use: Rheological, Thermal, and Physicochemical Evaluation

**DOI:** 10.3390/ma19020289

**Published:** 2026-01-10

**Authors:** Roberto Grosso, Fátima Díaz-Carrasco, Elena Vidal-Nogales, M.-Violante de-Paz, M.-Jesús Díaz-Blanco, Elena Benito

**Affiliations:** 1 Departamento de Química Orgánica y Farmacéutica, Facultad de Farmacia, Universidad de Sevilla, C/Prof. García González, 2, 41012 Sevilla, Spain; rgrosso1@us.es (R.G.); fdiaz4@us.es (F.D.-C.); ebenito@us.es (E.B.); 2 Department of Chemical Engineering, Physical Chemistry and Materials Science, University of Huelva, Campus “El Carmen”, 21071 Huelva, Spain; dblanco@diq.uhu.es; 3 Pro2TecS–Product Technology and Chemical Processes Research Centre, University of Huelva, Campus “El Carmen”, 21071 Huelva, Spain

**Keywords:** interpenetrating polymer network, gelatin, Diels–Alder, biopolymer, tissue engineering, hydrogel

## Abstract

**Highlights:**

**What are the main findings?**

- Gelatin-based IPN showed enhanced mechanical and thermal stability via Diels–Alder chemistry;
- Optimal mechanical performance occurred at ~3% (*w*/*v*) gelatin and low–moderate crosslinking;
- Excessive crosslink density caused network heterogeneity and reduced moduli and viscosity;
- Systems remained stable at physiological temperature.

**What are the implications of the main findings?**

- Gelatin content enables controlled and tunable degradation;
- Tunable properties allow application-specific design for tissue engineering.

**Abstract:**

Tissue engineering is a multidisciplinary field that aims to address tissue and organ failure by integrating scientific, engineering, and medial expertise. Gelatin is valued in this field for its biocompatibility; however, it faces thermal and mechanical weaknesses that limit its biomedical utility. This work proposes a strategy for improving gelatin properties by fabricating semi-interpenetrating polymer networks via *in situ* Diels–Alder crosslinking within gelatin colloidal solutions. Ten systems with variable polymer concentrations (2–4%) and crosslinking degrees (2–5%) were prepared and characterized. Rheological analysis revealed that elastic modulus, zero-shear viscosity, and complex viscosity were substantially enhanced, being especially dependent on the crosslinking degree, while critical strain values mostly depended on gelatin concentration. The incorporation of a synthetic Diels–Alder-crosslinked network also improved the thermal stability of gelatin hydrogels, particularly at physiological temperatures. Additionally, these systems exhibit favorable buoyancy, swelling and biodegradation profiles. Collectively, the resultant hydrogels are cytocompatible, solid-like, and mechanically robust, allowing for further tunability of their properties for specific biomedical uses, such as injectable matrices, load-bearing scaffolds for tissue repair, and 3D bioinks.

## 1. Introduction

Tissue engineering relies on the use of biomaterials to replace or assist the regeneration of diseased or damaged tissues [1,2]. These biomaterials can act as scaffolds and support cell growth and tissue regeneration. Among various natural polymers, gelatin has attracted growing interest in recent years. It is obtained from the partial hydrolysis of collagen, which yields a heterogeneous blend of peptide-based fragments with diverse molecular weights. Consequently, gelatin cannot be classified as a single, well-defined chemical compound, but rather a complex mixture of amino acid sequences linked by peptide bonds to form low molecular weight (around 15,000 Da) that can also aggregate into larger complexes (200,000 to 300,000 Da) [3,4].

Traditionally, its applications were largely confined to the food, art and cleaning industries [5,6], but more recently, gelatin has also emerged as one of the most widely utilized biomaterials in tissue engineering, thanks to its biocompatibility, biodegradability, low cost, non-toxicity, high swelling performance, and non-immunogenicity [7]. In addition, its capacity to promote cell adhesion, proliferation and differentiation, along with its susceptibility to enzymatic degradation by endogenous enzymes (e.g., matrix metalloproteinases) without inducing an immune response, have led to its exploration as a key component in the development of drug delivery matrices, as a matrix for the synthesis and immobilization of nanoparticles of various chemical compounds, and as a valuable source for the fabrication of protein nanomaterials [6]. Overall, gelatin is regarded as a highly promising candidate for scaffold fabrication, 3D bioprinting, drug delivery, and wound healing [1,8,9].

Nonetheless, colloidal solutions of gelatin exhibit critical limitations when employed in tissue engineering. First, the degradation rates of gelatin hydrogels may not align with the rates of tissue regeneration, leading to suboptimal outcomes in tissue engineering applications [10]. Second, gelatin-based hydrogels often display inadequate mechanical strength, which can restrict its application in load-bearing tissues when used by itself [10,11]. And last, it is prone to thermal degradation, undergoing sol-like to liquid-like transitions that are temperature-dependent (particularly around physiological temperatures) [1,12], which can compromise its structural integrity and functionality in drug delivery systems (especially when the application requires sustained exposure to elevated temperatures) [7]. All in all, these issues hinder the effectiveness and applicability of gelatin-based systems, compromising its ability to form stable 3D structures and hydrogels for *in situ* applications [1] (even when the synthesis of gelatin-based hydrogels has already been tackled) [13,14,15,16,17].

One of the most common strategies to overcome this limitation is the use of crosslinking. Widely described methods involve the use of ultraviolet light or microwave energy to rearrange the amino acid sequences in gelatin; however, this approach often lacks effectiveness when controlling crosslinking density and may generate cytotoxic by-products, limiting their biomedical applicability [18,19,20]. Gelatin can also be reinforced by direct covalent crosslinking involving its amino groups. In particular, the crosslinking behavior and gel properties of 3 wt% type A and type B gelatin catalyzed by microbial transglutaminase (MTG) have been studied. This enzyme catalyzes the formation of covalent bonds between γ-carbonyl and ε-amino groups of glutamine and lysine substrates, respectively [21]. Increasing crosslinking density via this technique modulates mechanical properties, swelling, and thermal stability [22]. In type B gelatin, covalent crosslinks induce partial protein polymerization, but physical crosslinking remains dominant because of its lower glutamine content, so the gel properties are largely unchanged even at high enzyme loadings. As a result, MTG treatment leads to clear differences in the response of type A and type B gelatin [22].

Other strategies involve crosslinking gelatin with (di)aldehydes, whose formyl groups react with free amino groups in gelatin via Schiff base formation. Glutaraldehyde (GLU) is the most widely used crosslinker in this context [23]; however, its application in biomedical contexts is limited by toxicity concerns. Glyceraldehyde (GAL) has been proposed as a more biocompatible alternative, although the underlying crosslinking mechanism remains to be fully elucidated. FE-SEM analysis indicates that GLU- and GAL-crosslinked hydrogels demonstrate diminished order and homogeneity of the gelatin structure at the micrometric scale [23]. At the nanoscale, GLU-crosslinked samples display network heterogeneities compatible with crosslinker-rich domains, whose size decreases with increasing GLU content. As anticipated, GLU-crosslinked hydrogels exhibit reduced deformations under a constant stress load, indicative of a more rigid network where crosslinking junctions impede chain rearrangement [23]. A related approach utilizes aldehyde-bearing cellulose nanowhiskers as multifunctional crosslinkers, resulting in gelatin hydrogels with significantly increased storage modulus and enhanced thermal stability compared to pristine gelatin [24].

Given this, the field has also responded to gelatin’s mechanical limitations by blending it with synthetic polymers or incorporating it into interpenetrating polymer networks (IPN) to enhance its properties. These systems aim to improve mechanical and thermal stability without sacrificing biofunctionality. Some examples included the combination of gelatin with cellulose [20], alginate [25], alginate and cellulose [26], 2-hydroxyethyl methacrylate [27,28] poly(vinylpyrrolidone) [29,30], acrylamide and itaconic acid [31], polyacrylamide [32], silk fibroid [33], polyhydroxyurethanes [34], hydroxyethyl cellulose and chitosan [35], or hyaluronic acid [36], among others. Many current approaches rely on only physical or ionic crosslinking to ameliorate mechanical properties of the mixture, which often result in poorly defined structures, limited mechanical performance, and batch-to-batch variability [37,38]. Furthermore, few systems allow for mild, cell-friendly crosslinking conditions suitable for *in situ* tissue regeneration or injectable systems. In this context, the Diels–Alder (DA) click reaction has emerged as a promising tool for gelatin-based hydrogel formation reinforced by a covalent 3D network. This [4+2] cycloaddition between dienes (e.g., furan) and dienophiles (e.g., maleimide) proceeds under physiological conditions, requires no catalyst, produces no by-products, and is reversible and bioorthogonal—thus making it compatible with natural processes in biological systems [39]. These features make it ideal for biomedical applications where crosslinking must occur *in situ* without harming encapsulated cells or tissues, such as cell scaffold formation. The resulting hydrogels exhibit high mechanical strength owing to the formation of two covalent bonds per diene–dienophile pair, effectively overcoming the limitations associated with physical or ionic crosslinking methods. Furthermore, this covalent architecture allows for precise tuning of the hydrogel’s mechanical properties by controlling the crosslinking density, enabling closer mimicry of native tissue mechanics [39,40,41].

In this work, we propose a novel strategy to address the mechanical and thermal limitations of pristine gelatin hydrogels by developing a semi-IPN composed of gelatin and a synthetic polymer formed via the DA reaction between furan and maleimide groups. These systems would offer several potential advantages over previous approaches: (a) bioorthogonal and cytocompatible crosslinking, enabling *in situ* gelation; (b) enhanced mechanical performance, due to the addition of a covalently crosslinked network; (c) compatibility with drug loading, enabling controlled therapeutic release; and (d) thermoreversibility, offering control over gelation and injectability. Our systems are expected to remain in a gel-like state at physiological temperatures, preventing transition into a sol-like phase upon heating (as typically observed with unmodified gelatin) and ensuring structural integrity and sustained performance *in vivo*. Nonetheless, the incorporation of DA-based crosslinking is expected to introduce a temperature-responsive reversibility at higher thermal thresholds at the same time. This feature would allow the hydrogel to undergo selective degradation via retro-DA reactions when exposed to localized heating, thus facilitating on-demand drug release and potential clearance of the material from the site. Such dual thermal behavior would provide a unique platform for precise control over both therapeutic delivery and post-application biodegradability.

To our knowledge, the use of DA-crosslinked IPN has not been extensively explored in gelatin-based systems aimed at tissue engineering applications. In this way, the resulting DA-crosslinked gelatin semi-IPN are anticipated to display improved physicochemical and rheological characteristics while reducing the presence of heterogeneities found with other crosslinking techniques. This would thus render them promising candidates for a plethora of biomedical uses tailored to specific property profiles achieved, i.e., (a) 3D printed scaffolds with tunable mechanical strength [14,42]; (b) injectable hydrogels offering protective cushioning for joint tissues [43]; or (c) therapeutic delivery systems, where bioactive molecules such as anti-inflammatory drugs are incorporated to aid tissue repair and reduce symptoms in degenerative diseases [44]. By addressing the main limitations of current gelatin-based materials (poor mechanical performance, and thermal instability) and introducing a versatile, biocompatible crosslinking strategy, this study contributes a new platform for the development of next-generation hydrogels in tissue engineering.

## 2. Materials and Methods

### 2.1. Materials and General Methods

Type I, type B gelatin from bovine skin (reference number: G9382-100G) was acquired from Aldrich Chemical Co. (Madrid, Spain). The remaining chemicals used in this work were also purchased from the same company.

Rheological tests and scanning electron microscopy (SEM), as well as freeze-drying procedures, were conducted at the CITIUS Service (University of Seville). Rheological measurements of the hydrogels were performed using a Discovery HR-3 rheometer (TA Instruments, New Castle, DE, USA), equipped with a Peltier temperature controller and a plate–plate geometry (diameter: 40 mm). The normal (axial) force was continuously monitored during all experiments. For all gel samples, the normal force remained low and stable, always below 10 N, and was zero in the absence of a sample. Images were recorded specifically at the Microscopy Laboratories by means of a field emission SEM, Zeiss EVO (Carl Zeiss Microscopy GmbH; Oberkochen, Germany), at an accelerating voltage of 5 kV using secondary electrons. Infrared spectra were recorded with a Bruker Invenio-X spectrometer (Bruker Optics GmbH; Ettlingen, Germany) equipped with ATR. FTIR spectra were collected within a wavenumber range of 4000 cm^−1^ to 400 cm^−1^, with a resolution of 4 cm^−1^. The background reference material used was air.

Data analysis was performed using Microsoft Office Excel 365. Unless stated otherwise, all experiments were conducted in triplicate. Statistical significance was assessed by one-way analysis of variance (ANOVA) using Excel’s built-in statistical tools, considering *p* < 0.05 as the threshold for significance.

### 2.2. Synthesis of IPN

For illustrative purposes, the procedure for the preparation of IPN G_2_-Xr_2_ (Table 1) was as follows: 200 mg of type I bovine skin gelatin (Polymer 2) and 5 mL of distilled water were vigorously mixed inside a vial and then stirred at 38 °C until homogenization. Next, the necessary amount of DMSO was added to reach a total solvent volume of 10 mL, considering that each monomer of Polymer 1 and the crosslinking agent were dissolved in this same solvent. Then, the monomers and crosslinking agent for Polymer 1 (freshly prepared according to the procedures described in the literature and pre-dissolved in DMSO) were sequentially added in the feed: the difurfuryl monomer disulfanediyl bis(ethane-2,1-diyl) bis[(furan-2-ylmethyl)carbamate] [45] (DF; 111 mg at a concentration of 350 mg·mL^−1^ in DMSO), the trifrufuryl crosslinking agent 2,3,5-tri-*O*-[(furan-2-ylmethyl)carbamoyl]-d-ribono-1,4-lactone [46] (TF; 2 mg at a concentration of 60 mg·mL^−1^ in DMSO), and the dimaleimide monomer 1,8-dimaleimide-3,6-dioxaoctane [47] (DM; 87 mg at a concentration of 100 mg·mL^−1^ in DMSO). Lastly, the mixture was stirred at 40 °C for 24 h.

The hydrogels differ from each other in two parameters: polymers concentration (2%, 3% or 4% *w*/*v* for both Polymer 1 and Polymer 2), and degree of Polymer 1 crosslinking (2.0%, 3.5% or 5.0%). The crosslinking degree was achieved by adjusting the percentage of functional maleimide groups (from the DM monomer) available to react with all furan rings in the TF crosslinker and the DF monomer. The crosslinking percentage indicates the maximum fraction of maleimide groups in the feed that can form DA adducts with furan groups from TF.

In Table 1, G denotes Polymer 2 (gelatin), *m* indicates the *w*/*v* concentration of Polymer 1 and Polymer 2 in the formed hydrogels, and *n* denotes the degree of crosslinking (Xr) of Polymer 1. Hydrogels 5 and 6 were intentionally formulated to fit the Box–Behnken design applied in the present study.

### 2.3. Characterization of IPN

The rheological properties of resulting IPN were analyzed and compared with their respective blanks (colloidal solutions of Polymer 2 at 2%, 3% and 4% *w*/*v* concentrations; 10 mL H_2_O-DMSO 1:1 *v*/*v*). To determine the linear viscoelastic range (LVR) and critical strain (${\dot{\gamma }}_{c}$) of the samples, strain sweep tests were carried out at 25 °C and a constant frequency of 2π rad·s^−1^, within a strain range of 0.02% to 100%. Later, frequency sweep tests were conducted within the previously determined LVR, using an angular frequency range from π/25 to 200π rad·s^−1^. These tests provided values for the viscoelastic moduli ($G^{\prime }$ and $G^{\prime \prime }$, corresponding to the elastic and viscous moduli, respectively), as well as the loss tangent [tan (δ) = $G^{\prime \prime }/G^{\prime }$]. Complex viscosity ($\eta^{*}$), zero-shear viscosity ($\eta_{0}$) and plateau modulus ($G_{N}^{0}$) values were also determined. The optimal plateau region for $G_{N}^{0}$ calculation was selected by considering frequencies between 1 and 100 rad·s^−1^, ensuring that no individual point within this range deviated by more than ±5% from the average value of the plateau. Regarding $\eta_{0}$, since direct measurement of viscosity at exactly zero shear rate is impractical, $\eta_{0}$ values were obtained by fitting the low-shear $\eta^{*}$ data to the Carreau model and extrapolating the result to a strain rate approaching zero.

Thermal degradation studies of selected systems were carried out by using oscillatory temperature ramp tests (heating temperature ramp from 25 °C to 65 °C; heating rate: ±2 °C/min; frequency: 2π rad·s^−1^; and cooling temperature ramp, equivalent from 65 °C to 25 °C) to investigate their behavior with temperature. The experiment was designed for 80 min of heating, followed by a 2 min stabilization period, and then 80 min of cooling.

Swelling tests were performed on 50 mg beads of freeze-dried IPN samples. The beads were placed into Eppendorf tubes at room temperature and distilled water was added incrementally until visual saturation was achieved. The weights of the dried beads used in the study ($W_{1}$) were then compared with their weights after swelling ($W_{2}$). The swelling index (SI) is defined as the number of times the system absorbs an amount of water equivalent to its weight (in percentage). The index is determined by Equation (1):

(1) SI=W2−W1W1 · 100

Buoyancy and degradability experiments were carried out by immersing 50 mg of freeze-dried IPN in 15 mL of simulated gastric fluid (SGF) [48]. For the buoyancy tests, a medium temperature was maintained at 37 °C and the samples were allowed to float freely. The parameters measured for the samples that floated were as follows: (a) floating lag time (FLT): refers to the time required for the IPN to rise to the surface and float; and (b) floating time (FT): refers to the total time during which the system remains in a state of floating prior to descending once more or undergoing complete disintegration.

Stability of IPN samples was assessed by exposure to different temperatures for varying periods of time: (1) 25 °C for 12 days; (2) 37 °C for 48 h; (3) 37 °C for 12 days; and (4) 65 °C for 24 h. Each subsequent condition was applied only if the material had not completely degraded during the preceding stage.

The microstructure of the freeze-dried systems was analyzed by SEM, and the evaluation was based on the interpretation of the obtained images.

### 2.4. Box–Behnken Experimental Design

To thoroughly assess the impact of two independent variables (gelatin concentration and degree of crosslinking) on specific rheological properties of the synthesized systems, a Box–Behnken experimental design was employed (CSS Statistica, StatSoft Inc.; Tulsa, OK, USA). For this study, three gelatin blanks with polymer concentrations of 2%, 3%, and 4% were also prepared for comparison purposes (Table 2).

The required number of experiments (*N*) was determined by Equation (2):

(2) $$N\;=\;2^{k}+2\cdot k+cp$$

where *k* represents the number of factors (variables) involved, and *cp* is the number of replicates of the central point.

Box–Behnken can be conceptualized as a cube, with a central point and the midpoints of the edges. When the independent variables are considered at three levels, a total of 10 experiments is required for each system (Table 2). In accordance with the prescribed methodology for implementing the experimental design method, two identical systems, designated as systems 5 and 6, were prepared.

To facilitate direct comparison of the coefficients and to visualize the effects of each independent variable on the rheological parameters studied, the values of the independent variables were normalized from −1 to +1 (Table 2) using Equation (3):

(3) Xn=X−X¯(Xmax−Xmin)2

where $X_{n}$ is the normalized value of independent variables; X is the absolute experimental value of the variable concerned; $\bar{X}$ is the mean of all fixed values for the variable in question; and $X_{max}$ and $X_{min}$ are the maximum and minimum values of the variable, respectively.

## 3. Results and Discussion

In this study, we assessed the preparation and characterization of IPN systems formed by *in situ* generation and crosslinking of a synthetic polymer (Polymer 1) within a colloidal solution of gelatin via DA reactions. The potential application of these matrices in tissue engineering will depend on how gelatin concentration and degree of crosslinking influence the physicochemical and rheological properties of these materials.

### 3.1. Synthesis of IPN

**Figure 1:**
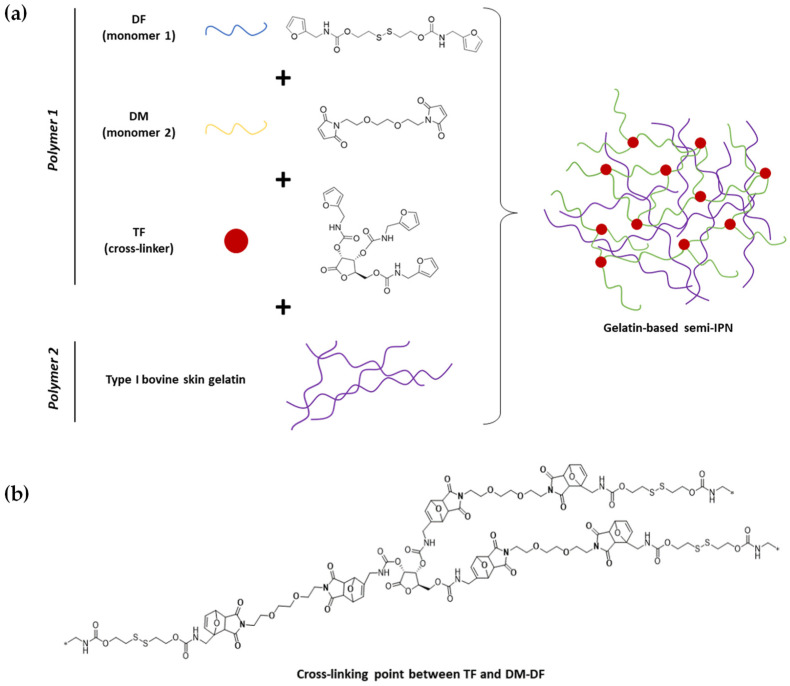
(**a**) *In situ* formation of the IPN by the growth of crosslinked Polymer 1 within a colloidal solution of Polymer 2 (gelatin), and (**b**) scheme of resulting crosslinking points. Asterisks represent polymer chain truncation for schematic representation.

A total of 10 gelatin-based IPN (Table 1) were successfully synthesized and thoroughly characterized. For the preparation of the hydrogels, the crosslinked Polymer 1 was synthesized via *in situ* DA reaction between DF and DM within a colloidal solution of gelatin (Polymer 2; H_2_O-DMSO 1:1 *v*/*v*). The weight ratio of the two polymers was 1:1 for all IPN. Formation of the 3D-Polymer 1 network was conducted at 40 °C under gentle stirring for 24 h. Polymer chains derived from the reaction between both monomers were interconnected to form a three-dimensional network around the gelatin, facilitated by the crosslinking agent TF, thus generating an IPN system (Figure 1a). The crosslinking point generates tricyclic systems formed by the reaction between the maleimide rings of DM and the furan rings of TF (Figure 1b).

It was observed that after 24 h at 40 °C under constant stirring, the IPN appeared as a viscous liquid that gels upon cooling to room temperature, with the time required for stabilization of their rheological properties varying between 2 and 5 days, depending on the polymer concentration.

IPN with a polymer concentration of 4% acquired a stable solid appearance after 48 h, whereas those with half the gelatin concentration required 5 days to gel. IPN with a 3% polymer concentration showed an intermediate appearance, as anticipated, and maintained consistent texture after 3 days at room temperature. Therefore, it was assumed that the DA reaction continues to progress over time between the chain ends of oligomers and polymers, with the process proceeding faster at higher polymer concentrations [46]. Thus, the rheological analyses of the hydrogels were conducted after a latency period of 5 days (at room temperature) after their preparation.

In previous work, we optimized the polymerization conditions for the DF–DM DA polymer [45]. The reaction proceeded efficiently in both organic and aqueous media, showing increased molecular weights in the studied temperature range (20–40 °C). Polymerization at 40 °C was selected for its effectiveness in the gelatin colloidal solution. As previously observed by ^1^H-NMR, the concentration of residual maleimide groups remained low and constant after 48 h, consistent with the step-growth nature of the process. It is expected that furan and maleimide moieties will remain as chain-end functionalities in the resulting DA polymers because equimolar consumption of maleimide and furan groups occurs.

**Figure 2:**
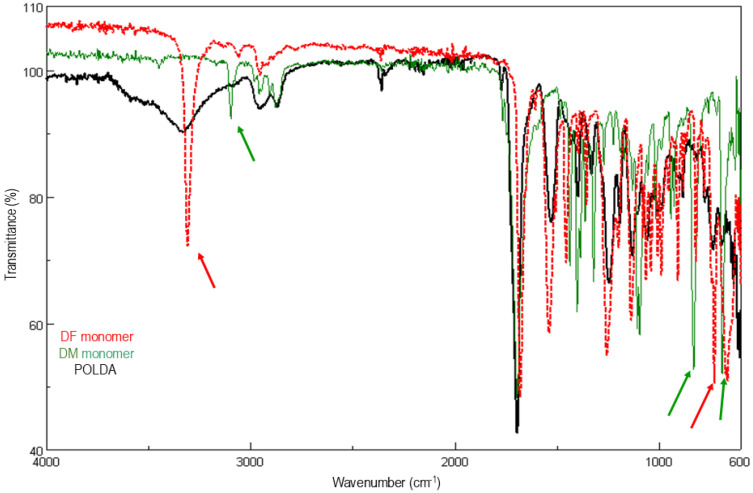
Overlay of FTIR transmittance spectra of POLDA (black), DM (green) and DF (red). The arrows indicate the most significant bands regarding the DA polymerization process.

In the present study, the complexity of the IPN system (arising from the 3D crosslinked network, the chemical heterogeneity of gelatin, and the solid-like hydrogel structure) limits the suitability of ^1^H-NMR analysis. Therefore, FTIR spectroscopy was employed as an effective and economical technique to monitor the DA polymerization within the IPN formation. Thus, to better visualize the formation of the DA network, the FTIR spectra of the DF and DM monomers and the resulting DA polymer (POLDA) were compared. The characteristic absorption bands and their evolution are summarized in Table 3 and Figure 2. The band at 3309 cm^−1^ can be monitored to study the evolution of polymerizations, showing a significant decrease in intensity (from DF), whereas those at 3098, 820, 832 and 692 cm^−1^ are nearly undetectable (from DM and DF).

**Figure 3:**
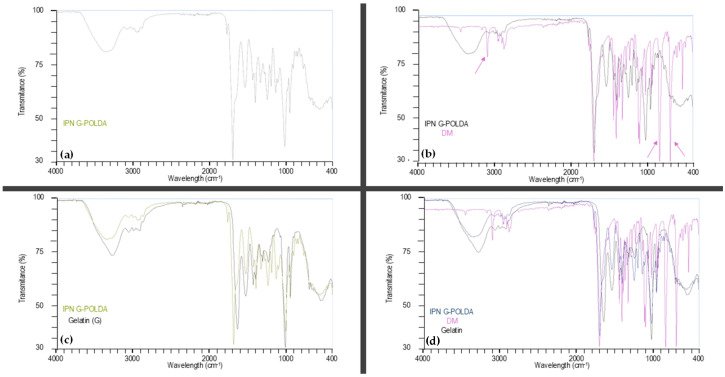
FTIR transmittance spectra of (**a**) IPN-G-POLDA, overlaid with (**b**) DM, (**c**) gelatin, and (**d**) DM and gelatin. The arrows in Figure 3b indicate the most significant bands regarding the DA polymerization process.

Figure 3 shows the FTIR transmittance spectra of IPN-G-POLDA (Figure 3a, referring to IPN G_4_-Xr_3.5_) and its overlays with the monomer DM, gelatin, and both DM and gelatin (Figure 3b, Figure 3c and Figure 3d, respectively). These figures facilitate the visualization and interpretation of POLDA formation within gelatin colloidal solutions. As outlined in Table 3, the initial bands identified for tracking IPN formation were 3309, 3098, 820, 832, and 692 cm^−1^. However, when gelatin is incorporated into the composition, the band at 3309 cm^−1^ (assigned to the N-H stretching vibration in DF) becomes unsuitable for monitoring the DA polymerization during IPN formation. This limitation arises because the signal is masked by the overlapping broad N–H and O–H bands from gelatin (Figure 2), centered around 3300 cm^−1^. Consequently, three of the remaining four diagnostic bands are associated with the DM monomer (as illustrated in Figure 3b), which validates its selection as the most suitable species for monitoring IPN formation. These three bands are significantly attenuated or become undetectable in the IPN FTIR spectrum (Figure 3a), confirming the formation of the POLDA network and, therefore, the successful generation of the IPN structure.

It is relevant to highlight that the hydrogels prepared have the potential to serve as smart drug carriers, too. This is mainly attributed to the presence of dynamic and responsive structural motifs within the network. In particular, one of the DA-network constituents, the DF monomer, contains disulfide linkages, which can confer sensitivity to reduced glutathione (GSH), a reducing agent that is overexpressed in cancer tissues [49,50]. This feature is especially attractive for the development of stimuli-responsive anticancer formulations. It is not expected that these disulfide linkages significantly react with the limited number of sulfhydryl groups present in gelatin, as the reduced mobility of both segments would strongly limit such interactions. However, if such interactions were to occur, the resulting material would contain disulfide linkages connecting the DA network and gelatin. These linkages could be reversibly cleaved under physiological GSH conditions, thereby maintaining the controlled drug-release behavior of the hydrogel system.

### 3.2. Rheological Characterization of IPN

The rheological properties of all prepared systems were studied through strain and frequency sweeps and compared to their respective controls. In all cases, $G^{\prime }$ predominated over $G^{\prime \prime }$ (Table 4), consistent with previous findings where additional components are incorporated to improve the mechanical performance of gelatin-based hydrogels [51]. In essence, most evaluated rheological parameters were significantly enhanced (*p* < 0.05) compared to those of the respective blanks (pristine gelatin colloidal solution; in some cases up to several orders of magnitude greater), confirming the expected boost in mechanical performance. This improvement is attributed to the greater structural stability and integrity of the IPN matrices. A similar trend has been observed in hybrid gelatin–alginate systems [52].

Some semi-IPN demonstrated significantly enhanced properties compared to blank gelatin controls, indicating increased network stiffness and viscoelastic stability resulting from covalent crosslinking and polymer interpenetration. The functionality of the semi-IPN is enhanced by the crosslink density of Polymer 1 and the gelatin concentration, which serve as the primary modulators of these mechanical enhancements. $G^{\prime }$ consistently exceeds $G^{\prime \prime }$ values across all samples, with tan (δ) ≤ 0.30, confirming the predominance of elastic, solid-like behavior with minimal energy dissipation. Moreover, at all the gelatin concentrations tested (from 2% to 4%), $G^{\prime }$ increased by two to three orders of magnitude compared to the blank controls, indicating enhanced physically and covalently crosslinked domains from the gelatin triple helix formation and 3D-DA network. The maximum value of $G^{\prime }$ was reached when the gelatin concentration was close to 3%, and the degree of crosslinking was low (around 2%). In any case, the values $G^{\prime }$ ≈ 14 kPa for G_3_-Xr_2_, $G^{\prime }$ ≈ 8 kPa for G_2_-Xr_2_ and $G^{\prime }$ ≈ 5 kPa G_4_-Xr_2_ were comparable to soft tissue targets like cartilage or adipose matrices. Previous studies have shown that gelatin methacrylate–silk fibroin semi-IPN of similar composition incorporate both physical and photoinduced crosslinks, with stiffness tuning ($G^{\prime }$ = 10^3^–10^5^ Pa) governing their compatibility with various tissues [53].

**Figure 4:**
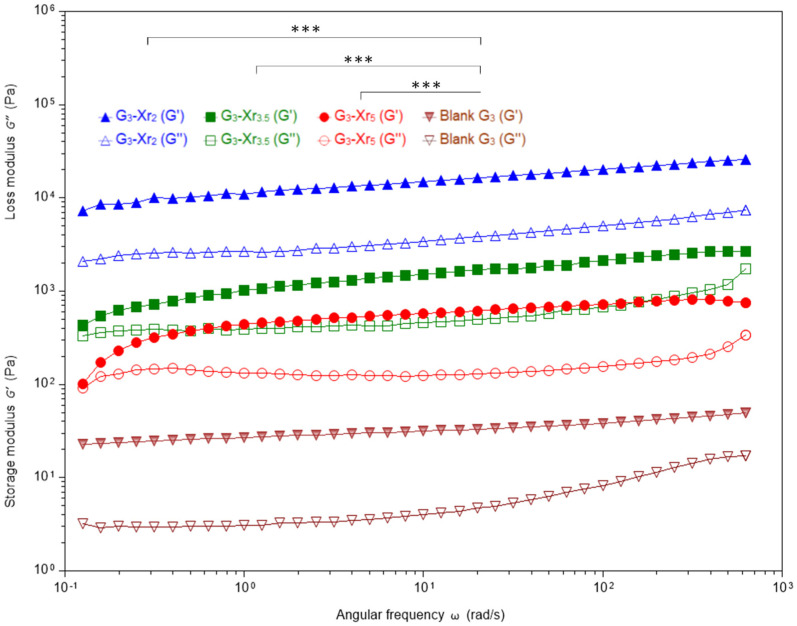
Frequency sweep (π/25 to 200π rad·s^−1^) of IPN with a gelatin concentration of 3%, and the corresponding blank. Filled symbols represent $G^{\prime }$, while open symbols represent $G^{\prime \prime }$. *** = *p* < 0.001.

**Figure 5:**
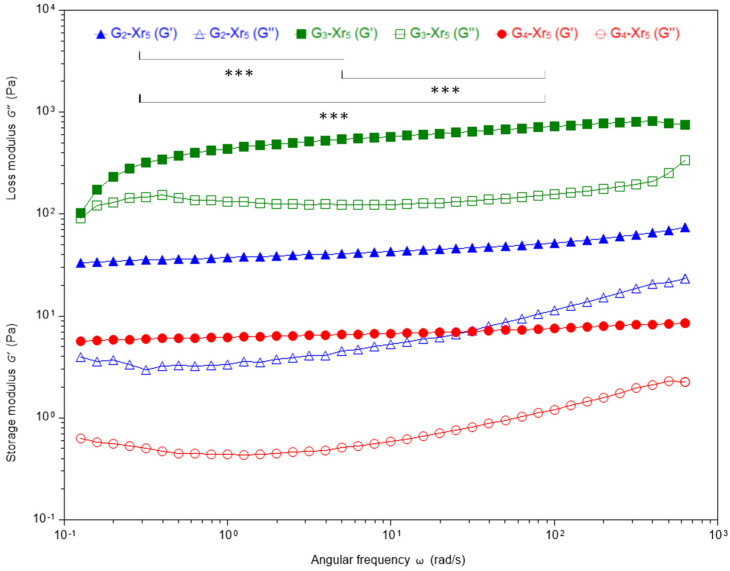
Frequency sweep (π/25 to 200π rad·s^−1^) of IPN with a crosslinking degree of 5%. Filled symbols represent $G^{\prime }$, while open symbols represent $G^{\prime \prime }$. *** = *p* < 0.001.

The frequency-sweep data displayed in Figure 4 and Figure 5 offer additional support for these observations. The first figure presents the frequency-dependent evolution of $G^{\prime }$ and $G^{\prime \prime }$ as a function of angular frequency for three semi-IPN with 3% gelatin compared to their blank counterparts, whilst the second one illustrates the frequency-dependent behavior of samples prepared at a constant crosslinking degree of 5%, tested over a range of π/25 to 200π rad·s^−1^. The $G^{\prime }$(ω) slopes remained low, indicating stable elastic networks unaffected by oscillatory frequency (a crucial aspect for biomedical reliability under cyclic loading). As reported by Carbajo-Gordillo et al. [34], gelatin-based IPN in DMSO/H_2_O mixtures exhibit similar resilience. The plateaued $G^{\prime }$ behavior is attributed to the coexistence of covalent DA networks and reversible hydrogen bonding.

It is noteworthy that G_3_-Xr_2_ presented a $G^{\prime }$ value ≈ 2.5 order of magnitude greater than that of the blank. A similar trend was observed for zero-shear viscosity. In any case, for all the gelatin concentration series (from 2% to 4%), the optimal rheological performance was attained at the lowest levels of crosslinking (Xr_2_ > Xr_3_ > Xr_4_; Table 4 and Figure 4).

As illustrated in Figure 5, both $G^{\prime }$ and $G^{\prime \prime }$ moduli exhibited non-monotonic trends in relation to gelatin content. A maximum was observed at 3% gelatin, followed by 2% gelatin content, while the lowest values were recorded for G_4_-Xr_5_. The decrease in moduli at 4% gelatin suggests the formation of heterogeneous microdomains, which hinder the formation of a continuous DA network and limit intermolecular hydrogen bonding among gelatin chains. In fact, in the field of IPN, it has been observed that elevated polymer concentrations can result in network heterogeneities and phase separation. For instance, Djabourov’s work details how high gelatin concentrations impede triple-helix formation and generate heterogeneous domains that disrupt modulus development [54]. Therefore, when comparing the systems’ formulations, the G_3_-Xr_5_ formulation demonstrates the highest $G^{\prime }$, exceeding that of G_4_-Xr_5_ by over three orders of magnitude.

A substantial enhancement was also observed in most plateau modulus and complex viscosity values when compared to those of blanks. $G_{N}^{0}$ is a measure of the material’s mechanical properties that reflects its elastic behavior in the rubbery plateau region, and is defined as the constant value of $G^{\prime }$ observed over a range of low-medium frequencies, before the material enters a regime of more pronounced viscous relaxation [55,56]. In the studied systems, $G_{N}^{0}$ varied from <10 Pa (blank G_2_ and G_4_-Xr_5_) to ~14.5 kPa (G_3_-Xr_2_), increasing markedly with low crosslinking and moderate gelatin concentration. Higher $G_{N}^{0}$ values indicate more robust 3D networks with well-distributed stress pathways, which in turn implies increased resistance to deformation and an enhanced ability to maintain shape under mechanical loads. However, excessive crosslinking (e.g., Xr = 5) drastically reduced $G_{N}^{0}$ (≈7 Pa for G_4_-Xr_5_), suggesting over-rigid domains that fracture prematurely. These trends mirror gelatin IPN behavior observed by Carbajo-Gordillo et al. [34], where $G_{N}^{0}$ was identified as a sensitive probe of entanglement density and microstructural strength.

The loss tangent, defined as tan (δ) = $G^{\prime \prime }/G^{\prime }$, reflects the phase lag between stress and strain, and indicates the ratio of energy dissipated to energy stored in the material. Most IPN registered tan (δ) ≤ 0.3, transitioning toward an elastic-dominated regime. Low values (0.08–0.18) reflect efficient energy storage and minimal damping, ideal for load bearing scaffolds exposed to repetitive stresses. Slightly higher values (0.23–0.31) seen in the G_3_-Xr*_n_* series highlight a viscoelastic compromise, suitable for injectable and dynamic matrices capable of transient deformation without structural loss. Comparable tan (δ) ranges (0.09–0.14) were observed in polyhydroxyurethane-gelatin semi-IPN, indicating optimized elasticity and mechanical recovery [34].

The critical strain values reported in Table 4 for the DA-crosslinked gelatin semi-IPN display a broad variation (from 0.43% in G_3_-Xr_3.5_ to ≈30% in G_4_-Xr_5_). Critical strain is described as the point at which the storage modulus decreases by 10% from its initial value [57]. At low gelatin content (between 2–3%), the IPN exhibited the smallest $\dot{\gamma_{c}}$ values, indicating that these systems are stiff and brittle, failing at minimal strain. Such behavior correlates with restrictive chain mobility and limited elastic recovery. This response is typical of dense dual-network hydrogels where the covalent phase dominates elasticity and minimizes reversible deformation before fracture [58]. In contrast, when the gelatin content increased to 4%, $\dot{\gamma_{c}}$ rose dramatically (up to values above 30% for G_4_-Xr_5_) indicating greater network plasticity and chain extensibility. Gelatin provides flexible physical junctions (triple helices and hydrogen bonds) that allow the matrix to accommodate higher deformation before breakdown. Similar improvements in strain tolerance associated with biopolymer reinforcement have been observed in semi-IPN composed of gelatin or collagen interpenetrated with synthetic polymers [22,59].

Within each gelatin series, the highest $\dot{\gamma_{c}}$ values are found for 5% of the crosslinking degree. This relationship is in line with the findings of Shrivastava et al. [60], who demonstrated that increasing crosslinking density in gelatin networks enhances fracture toughness and deformability by repeatedly arresting cracks. The enhancement was attributed to the synergy between covalent crosslinks and reversible supramolecular bonds, which function to dissipate stress and avert catastrophic cracking.

$\eta_{0}$ reflects the viscosity that a material exhibits under very low shear rates or minimal disturbance, representing its intrinsic resistance to deformation under quasi-static (near-zero) flow conditions [61]. Thus, it can serve as a sensitive indicator of polymer entanglement density, molecular mobility, and injectability potential under low-stress conditions. Regarding $\eta_{0}$ values reported in Table 4, they exhibit pronounced differences among the studied systems, demonstrating how viscosity is also governed by both crosslinking density and gelatin concentration. In the case of plain gelatin colloidal solutions, it has been found that increasing gelatin concentration results in greater values of $\eta_{0}$ ranging from 49 Pa·s (2%) to 1187 Pa·s (4%), which is indicative of weak physical gels dominated by transient hydrogen bonds at 2–3% gelatin concentration. In general, enhanced $\eta_{0}$ is observed for the DA-crosslinked semi-IPN, with maxima attained when the lowest crosslinking degrees are targeted during IPN preparation (for example, 21.0 kPa·s, 20.1 kPa·s, and 10.2 kPa·s for G_2_-Xr_2_, G_3_-Xr_2_ and G_4_-Xr_2_, respectively). This increase is attributed to the formation of extended, entangled gelatin-rich domains that interact through hydrogen bonding while being only loosely constrained by the covalent DA junctions, which effectively promote high flow resistance. Similar trends have been reported in semi-IPN where gelatin or collagen networks are interlinked with covalent backbones, resulting in viscosity increments of several orders of magnitude [59,62].

On the contrary, $\eta_{0}$ decreased significantly when crosslinking density exceeded optimal levels. For instance, at 3% gelatin, the viscosity dropped from ≈20.1 kPa·s (Xr_2_) to ≈1.1 kPa·s (Xr_5_). This decline indicates that overcrosslinking generates microphase domains that hinder effective stress transfer. Documented cases of this behavior have also appeared in hydrogels where excessive covalent junctions have been shown to result in microheterogeneous structures with reduced long-range viscosity and brittleness [63]. In contrast, low and moderate crosslinking (2.0–3.5%) promoted a synergistic reinforcement between the covalent and reversible gelatin phases, yielding pseudoplastic materials with high zero shear viscosity yet acceptable deformability. This is key for injectable formulations and 3D printing bioinks where reversible flow is desirable during processing.

As for the matter of complex viscosity values, it was observed that this parameter increased in IPN compared to the blanks. This is due to a greater number of molecular interactions, which leads to a higher probability of chain entanglements within the colloidal system [64]. All samples exhibited shear-thinning behavior (non-Newtonian fluids), characterized by a decrease in viscosity with increasing shear rate. It was determined that values of $\eta_{1}^{*}$ close to and above 1000 Pa·s were obtained for IPN with all gelatin concentrations and low crosslinking degrees. These values are comparable to those of molten polymers and up to 10 times higher than the complex viscosity reported for molasses (100 Pa·s).

In summary, it is essential that the plateau modulus be high to ensure system stability and elasticity in tissue engineering applications, with low values of tan (δ) being desirable [55]. In the case of considering their use as a substitute for synovial fluid in joint injuries, a high complex viscosity is required. These results suggest that the studied IPN have the potential to be used in different tissue engineering applications due to their superior mechanical properties and their non-toxic nature.

### 3.3. Box–Behnken Analysis

To deepen the analysis of the prepared systems, the effects of both the gelatin concentration and the crosslinking degree of Polymer 1 (independent variables) on key rheological parameters [critical strain, elastic modulus at 2π rad·s^−1^ (1 Hz), tan (δ) at 2π rad·s^−1^ (1 Hz), zero-shear viscosity, and complex viscosity at 2π rad·s^−1^ (1 Hz); dependent variables] were studied. This evaluation was conducted using a Box–Behnken model design, where the behavior of the selected rheological parameters was modeled as functions of the independent variables. The resulting equations are presented in Table 5.

**Figure 6:**
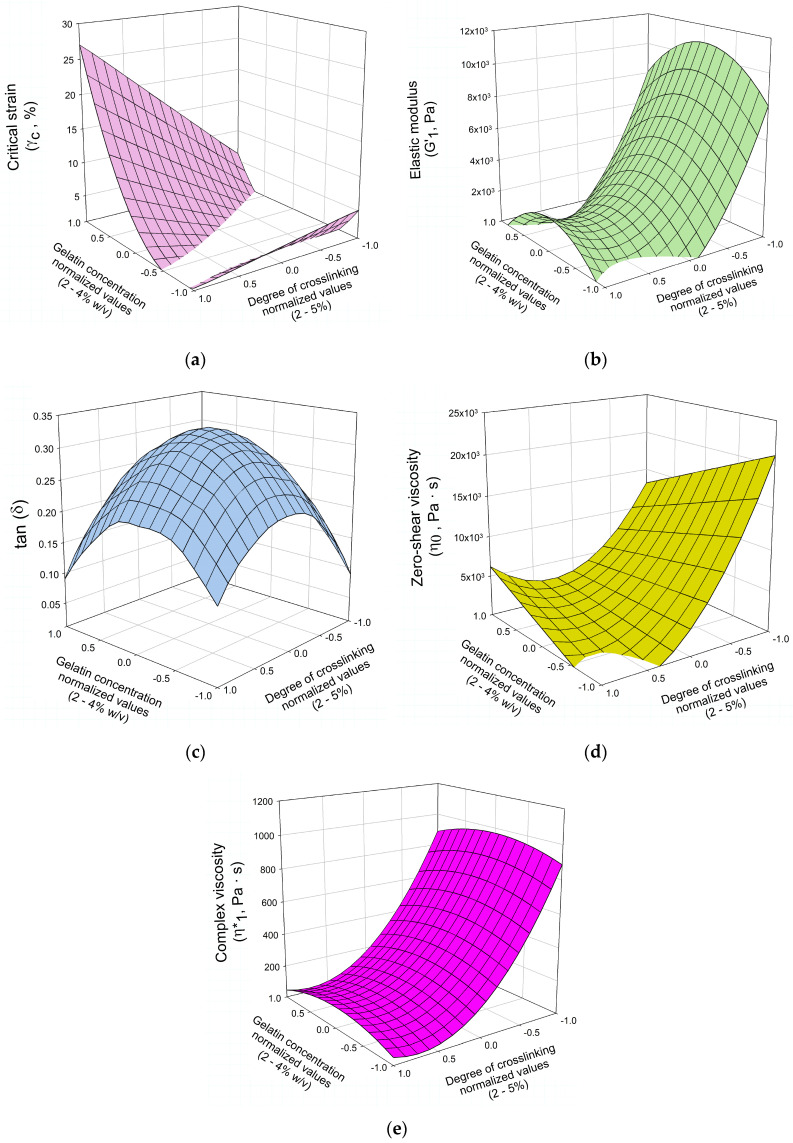
Response surfaces for (**a**) critical strain (%); (**b**) elastic modulus at 2π rad·s^−1^ (1 Hz; Pa); (**c**) tan (δ) (${G^{\prime \prime }}_{1}/{G^{\prime }}_{1}$); (**d**) zero-shear viscosity (Pa·s); and (**e**) complex viscosity at 2π rad·s^−1^ (1 Hz; Pa·s) of the prepared IPN.

To facilitate the understanding of these functional relationships, the equations are represented graphically in Figure 6 [critical strain (Figure 6a), elastic modulus at 2π rad·s^−1^ (1 Hz; Figure 6b), tan (δ) at 2π rad·s^−1^ (1 Hz; Figure 6c), zero-shear viscosity (Figure 6d), and complex viscosity at 2π rad·s^−1^ (1 Hz, Figure 6e)]. This representation allows for a more intuitive interpretation of the dependence between the rheological variables, and gelatin concentration and crosslinking degree of Polymer 1 in the analyzed systems.

In general, a strong dependence of the gelatin semi-IPN’s mechanical behavior was observed on both the crosslinking degree of Polymer 1 and the overall gelatin concentration. These parameters modulate the balance between elasticity, viscosity, and deformation tolerance of the hydrogels, which directly influence their suitability for biomedical uses such as injectable matrices and 3D scaffold materials.

**Figure 7:**
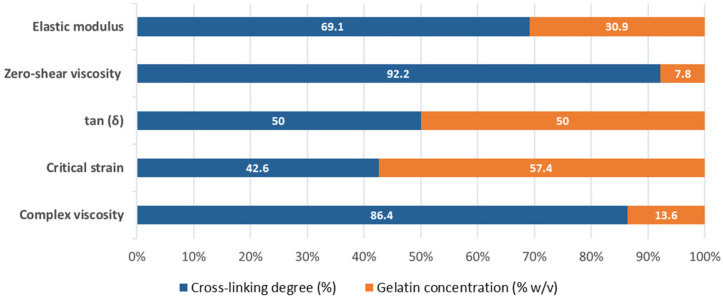
Relative influence (%) of the crosslinking degree (%) and gelatin concentration (% *w*/*v*) on the rheological parameters studied [elastic modulus, zero-shear viscosity, tan (δ), critical strain, and complex viscosity].

Specifically, the comparison of the rheological properties of the obtained IPN revealed that the degree of crosslinking had a greater influence on three of the five dependent variables, being decisive for $G^{\prime }$, $\eta_{0}$ and $\eta_{1}^{*}$. However, when analyzing $\dot{\gamma_{c}}$ values, gelatin concentration was the most influential independent variable (almost 60%). Influence on tan (δ) is similar for both parameters. Figure 7 details the relative influence (%) of both independent variables on analyzed rheological properties. Regarding the elastic modulus of the studied IPN hydrogels, $G^{\prime }$ increases with higher gelatin concentration but decreases as the degree of crosslinking rises (Figure 6b). The relative influence of each independent variable on $G^{\prime }$ was not the same, with degree of crosslinking accounting for 69.1% of the variation in the elastic modulus, while the gelatin concentration explains the remaining 31.9%, as evidenced by the relative slopes of the curves in the graph. Anyhow, the effect of concentration on $G^{\prime }$ became more pronounced at lower crosslinking degrees. This non-linear behavior indicates that both independent variables do not act independently but rather combine to amplify the mechanical response of the network. Furthermore, the sensitivity of $G^{\prime }$ to changes in gelatin concentration increased when the crosslinking degree was reduced, as demonstrated by the steeper slope of the response surface at low crosslinking levels. This underscores the necessity for concurrent optimization of both parameters to attain the desired mechanical stiffness.

Figure 6c illustrates the response surface resulting from Box–Behnken analysis of tan (δ) as a function of these two independent variables, showing low synergy between both on the viscoelastic balance of the network. The maximum tan (δ) value (0.31) is reached at intermediate values of the independent variables, i.e., at 3% gelatin concentration and 3.5% of crosslinking degree. Conversely, the minimum tan (δ) values are found at the extremes of the experimental space, both at low and high levels of both variables, respectively. Regarding the relative influence of each factor, it is observed that both the crosslinking degree and the gelatin concentration account for the same influence on the loss tangent (50%).

Box–Behnken analysis of the critical strain values (Table 5; Figure 6a) supports the trends described in the previous section regarding this variable and revealed a clear synergistic interaction between the independent variables. This relationship is evidenced by the distinctly positive slope of the response surface along the crosslinking degree axis and becomes more pronounced as the gelatin concentration increases. In this case, the relative influence of each independent variable is opposite to that observed for the rest of the analyzed rheological parameters, with gelatin concentration exerting a greater effect (57.4%) than the degree of crosslinking (42.6%).

From a biomedical standpoint, the variation in critical strain indicates tunability between rigid and flexible mechanical profiles. Low-$\dot{\gamma_{c}}$ IPN (≤1%) are well-suited for load-bearing implants or bone-mimetic scaffolds, where rigidity and dimensional integrity are paramount. High-$\dot{\gamma_{c}}$ hydrogels (≥10%) are advantageous for tissue fillers, injectable matrices, and soft-tissue scaffolds, requiring deformation without loss of cohesion. According to Li and Mooney [62], hydrogels with critical strain values above 10% exhibit superior *in vivo* mechanical adaptability, minimizing fracture under physiological loading and ensuring structural persistence at the defect site. The broad range of $\dot{\gamma_{c}}$ values observed in these DA-crosslinked gelatin semi-IPN indicates that it is possible to engineer materials with a spectrum of deformation limits by modulating polymer concentration and network crosslinking. These materials can span from brittle, high-modulus scaffolds to ductile, injectable architectures. This versatility ensures the ability to meet distinct biomedical mechanical demands.

Next, Figure 6d presents the response surface of zero-shear viscosity as a function of crosslinking degree and gelatin concentration, offering more insights into the interplay between polymer chain mobility, network connectivity, and overall flow resistance. In any case, the model equation for $\eta_{0}$ (Table 5) and its response surface representation (Figure 6d) support the aforementioned observations, clearly demonstrating the synergistic interaction between the two independent variables. Specifically, a reduction in crosslinking degree combined with an increase in gelatin concentration (up to approximately 3%) leads to a synergistic increase in zero-shear viscosity. This indicates that the material’s flow resistance improves when a higher polymer chain density (higher gelatin concentration) is paired with effective connectivity (low to medium crosslinking), resulting in a more robust and homogeneous network. Additionally, the influence of each independent variable differs substantially, with gelatin concentration exerting a lower effect (7.8%) compared to the crosslinking degree, which has a larger impact (92.2%).

From a functional standpoint, high $\eta_{0}$ values impart mechanical stability and resistance to dilution, which is critical for *in situ* forming gels intended for injectable tissue fillers, drug reservoirs, and cartilage repair matrices. Crosslinked gels with $\eta_{0}$ > 10^3^ Pa·s behave as viscoelastic solids, ensuring local retention after injection while maintaining sufficient flowability under shear [62,65]. Conversely, intermediate viscosities ($\eta_{0}$ ≈ 10^2^–10^3^ Pa·s) may be ideal for a wide range of clinical applications, such as bioinks or periarticular lubricating gels. In these cases, the material’s shear responsiveness ensures smooth injection and rapid structural recovery.

Lastly, Figure 6e shows Box–Behnken analysis results for complex viscosity, revealing a similar profile as the one found for ${G^{\prime }}_{1}$ (Figure 6b). The point of maximum complex viscosity (≈1295 Pa·s) is located in the central region of the experimental space, with gelatin concentrations of around 3% and low crosslinking levels (approximately 2%).

All in all, the Box–Behnken analysis further confirmed that all IPN showed significantly improved mechanical properties compared to pure gelatin colloidal solutions, with elastic modulus, zero-shear viscosity, and complex viscosity mainly influenced by crosslinking, while critical strain was more affected by gelatin concentration. Loss tangent was similarly affected by both variables. Optimal mechanical performance was achieved at intermediate gelatin levels and low crosslinking degrees.

### 3.4. Thermal Evaluation of IPN

Another objective of the characterization of IPN was to determine the thermal stability of the synthesized materials and compare them with gelatin colloidal solutions. Thermal studies eventually revealed that the incorporation of the DA-crosslinked synthetic 3D network effectively confers thermal stability to pristine gelatin.

**Figure 8:**
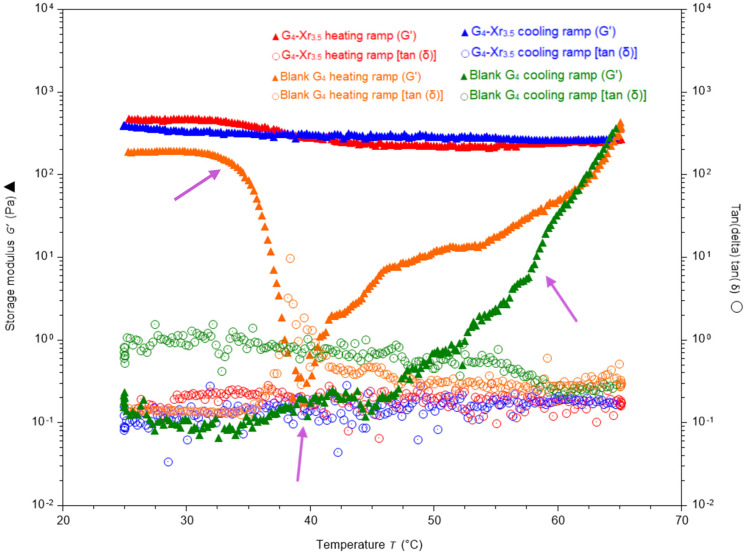
Variation in the storage modulus ($G^{\prime }$) and tan (δ) with temperature during heating and cooling ramps at 2 °C/min (25–65 °C, 1 Hz) for the IPN G_4_-Xr_3.5_ and its blank (4% gelatin). Purple arrows indicate key temperature points identified in the thermal analysis.

To do so, a comparative thermal study of IPN G_4_-Xr_3.5_ and its corresponding blank (colloidal solution of gelatin at 4% *w*/*v*) was performed, and its findings are presented in Figure 8 and Table 6. In the heating ramp, it is remarkable to observe how G’ values from the control sample experienced a significant decrease at physiological temperature (ranging from 32.14 °C to 38.15 °C, with a decrease in the storage modulus from 178.4 Pa to 0.7 Pa, respectively). The most pronounced drop in $G^{\prime }$ occurred at 40.37 °C, representing a decrease of ≈354 times compared to the first recorded $G^{\prime }$ value (at 25 °C). Meanwhile, tan (δ) underwent a transition from a gel-like to a sol-like behavior within the mentioned temperature range, with values ranging from 0.14 to 3.27. When the temperature exceeded 40 °C, $G^{\prime }$ exhibited a gradual recovery, reaching its initial value (i.e., the value prior to the onset of the experiment, at t=0) of 183 Pa at 64.03 °C. Overall, when comparing the initial value of $G^{\prime }$ (t=0) to the final value obtained after completion of the cooling ramp, a significant drop in $G^{\prime }$ could be observed (approximately three orders of magnitude, compared to only about 16% of the initial value for G_4_-Xr_3.5_), implying a complete structural breakdown of the system following the assay. These results demonstrate the protective effect exerted by the polymeric matrix of Polymer 1 against thermal degradation in gelatin-based hydrogels.

Nevertheless, tan (δ) increased to 0.38 at 64.03 °C, relative to its initial value of 0.15. Although it did not revert to the original value, these findings suggest the formation of a new viscoelastic hydrogel network, distinct from that observed at 25 °C. It is important to note that during the cooling ramp, $G^{\prime }$ values of the pristine gelatin control decreased from 361.7 Pa to 0.15 Pa (from 65 °C to 25 °C), and tan (δ) reached values above 1. This resulted in the loss of the gel-like nature of the sample with no recovery after cooling, demonstrating irreversible loss of elastic response due to gelatin denaturation [66].

In contrast, the gelatin-based IPN G_4_-Xr_3.5_ demonstrated consistent rheological properties, exhibiting only a slight—and later reversible—change in $G^{\prime }$ and tan (δ) within the 32–45 °C range during the heating ramp. In contrast to the gelatin blank, this IPN demonstrated exceptional resilience, maintaining its elasticity over a wide range of physiological and tested temperatures (up to 65 °C, Table 6). During the cooling experiment, $G^{\prime }$ recovered and tan (δ) improved to values better than those observed in the original sample [from tan (δ) = 0.14 to tan (δ) = 0.09]. This confirms that the thermoresponsive gel–sol transition of gelatin is suppressed in the IPN formulation, likely due to chemical crosslinking and polymer network reinforcement. Therefore, it can be stated that the thermal instability of gelatin hydrogels around physiological temperature is mitigated when interlaced with the DA-crosslinked Polymer 1 network, effectively forming an IPN more stable than the blank sample. It is also relevant to note that thermal degradation linked to DA polymers has not been observed within this temperature range [45].

### 3.5. Swelling, Degradation, Morphology and Application-Oriented Design of IPN

Swelling capacity and buoyancy are desirable characteristics for certain biomedical applications such as oral controlled-release, wound dressing or tissue engineering scaffolds [67,68,69]. For this reason, these parameters were determined for selected IPN after being freeze-dried (Table 7). Swelling tests showed a moderate swelling capacity across all systems. Hydrogels with the lowest gelatin concentration (2%) absorbed the least amount of water, increasing their initial weight by approximately 1.5 times. As the polymer concentration increased, the swelling capacity also increased, reaching a swelling index of 366% in the case of the IPN G_4_-Xr_5_, which corresponds to an almost fourfold increase in mass relative to its initial weight. On the other hand, when fixing the concentration of gelatin to 2–3%, it was observed that the highest swelling index corresponded to systems with the highest degree of crosslinking, which is consistent with other published studies [70].

Buoyancy assays revealed that all systems were able to float from the beginning of the test, presenting an initial floating lag time of 0 min (FLT_1_ = 0). Most IPN exhibited prolonged floating times [between 4 and 60 min (FT_1_)] and even the ability to resurface after temporary sinking. In this way, all IPN (except for G_3_-Xr_5_) exhibited a second floating lag time (FLT_2_), meaning they resurfaced after sinking and stayed afloat for a cumulative time ranging from 2.4 h to over 6 h (Table 7). Out of all the samples analyzed, the one that showed the longest buoyancy time was G_3_-Xr_3.5_, which remained afloat for more than 6 h (FT_2_ > 360 min).

Regarding degradation studies under mild conditions, none of the analyzed systems showed degradation signs after incubation at 25 °C for 12 days, nor after incubation at 37 °C for 48 h (Table 7). Only the IPN with 4% *w*/*w* gelatin concentration degraded completely after 12 days at 37 °C, while IPN with a 3% polymer concentration exhibited only partial degradation (showing some turbidity in the incubation medium). Lastly, those with the lowest gelatin concentrations remained mostly intact until reaching stage 4 (where degradation was accelerated by subjecting them to a temperature of 65 °C), demonstrating the highest structural stability. Even under these conditions, complete degradation of G_2_-Xr_2_ and G_2_-Xr_5_ occurred only after 2 and 3 days, respectively. These results are consistent with expectations, as gelatin is soluble in aqueous media at temperatures above 37 °C [70]. Therefore, the higher the gelatin content in the systems, the more easily they disintegrated. In addition to higher temperatures promoting gelatin dissolution, the retro-DA reaction occurred in Polymer 1 during the heating of the IPN, as previously observed [45], leading to the global disintegration of the systems.

**Figure 9:**
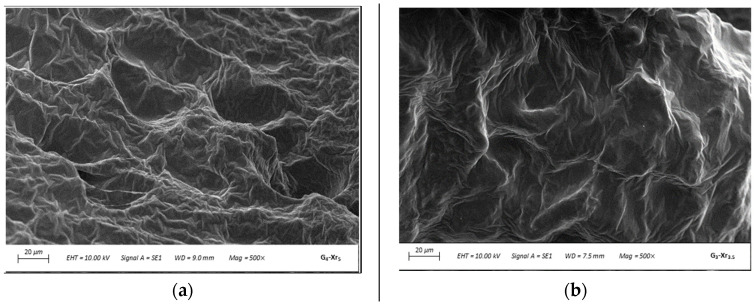
Micrographs of the IPN (**a**) G_4_-Xr_5_ and (**b**) G_3_-Xr_3.5_ (magnification: 500×).

Next, the morphology of freeze-dried IPN was analyzed by SEM. The micrographs shown in Figure 9 for the IPN G_4_-Xr_5_ and G_3_-Xr_3.5_ reveal the compact appearance of the samples, with surfaces that are non-porous and rough, and feature deep folds, which is consistent with previously published studies [71]. The most irregular surface is observed for IPN G_4_-Xr_5_, which exhibits the greatest swelling capacity due to its larger surface area [72].

To conclude this section and highlight the essential findings, Table 8 displays an integration of the rheological responses [critical strain, zero-shear viscosity, plateau and storage moduli, and tan (δ)], which permits an evidence-based evaluation of how each IPN formulation would perform in biomedical settings. These properties correlate directly with elasticity, flow behavior, resistance to mechanical fatigue, and recovery after deformation, all core factors defining the functional use of hydrogels in medical applications such as scaffolds, injectable systems, wound dressings, and stimuli-responsive matrices [59].

All in all, the most promising systems seem to be those of the G_3_ and G_4_ series (Table 8). On the one hand, the G_3_-Xr_2_ system combines the highest plateau and storage moduli with sufficient flexibility [tan (δ) = 0.23]. These values align perfectly with scaffolds designed for bone, cartilage, or tendon engineering, where elasticity must coexist with load support. On the other hand, G_3_-Xr_3.5_ is softer and may be more suitable for adipose or dermal scaffolds. G_4_ IPN show outstanding strain tolerance and viscoelastic control, with G_4_-Xr_2_ demonstrating the highest mechanical resilience ($G^{\prime }\;$> 5 kPa) and adequate tan (δ) [0.18]. This allows the combination of solid-like stability and elasticity comparable to native cartilage or nerve tissue matrices in just one system, making it suitable for injectable cushioning gels and 3D-printed scaffolds. Meanwhile, G_4_-Xr_3.5_ retains adequate deformability and could be advantageous as printable bioink.

## 4. Conclusions

In the present study, semi-interpenetrated polymeric networks (semi-IPN) were successfully prepared by crosslinking a synthetic polymer (Polymer 1), composed of disulfanediyl bis(ethane-2,1-diyl) bis[(furan-2-ylmethyl)carbamate] (DF) and 1,8-dimaleimide-3,6-dioxaoctane (DM), within a colloidal solution of a natural biopolymer (gelatin; Polymer 2) via Diels–Alder (DA) reaction, using 2,3,5-tri-*O*-[(furan-2-ylmethyl)carbamoyl]-d-ribono-1,4-lactone (TF) as a crosslinking agent. It was demonstrated that these semi-IPN provide substantial enhancements in mechanical, rheological, and thermal performance compared with pristine gelatin hydrogels. The combined effects of gelatin concentration and crosslinking degree enable precise tuning of stiffness and deformability across a range relevant to soft tissue and load-bearing biomedical applications.

Rheological analysis revealed that all formulations exhibit predominantly elastic, solid-like behavior with markedly increased storage modulus, plateau modulus, zero-shear viscosity, and complex viscosity relative to gelatin controls. Some formulations also maintain shear-thinning profiles that are favorable tissue engineering processes. The optimal mechanical performance was generally achieved at an intermediate gelatin content (approximately 3% *w*/*v*) and low to moderate crosslinking. Under these conditions, G_3_-Xr_2_ and related systems reached stiffness levels comparable to native soft tissues, yet they retain sufficient viscoelasticity for dynamic loading. Conversely, excessive crosslink density promotes network heterogeneities, leading to reduced moduli and viscosity.

The Box–Behnken analysis confirmed that crosslinking degree predominantly controls elastic modulus and viscosity, whereas gelatin concentration exerts a stronger influence on critical strain. Collectively, these factors allow the transition from brittle, high-modulus scaffolds to more ductile, highly deformable networks. Most IPN display low tan (δ) values, indicating efficient energy storage and suitability for load-bearing scaffolds. Slightly higher tan (δ) systems within the G_3_-Xr_n_ series offer a viscoelastic compromise well suited to injectable matrices and bioinks.

Thermal studies demonstrated that incorporating the Diels–Alder-crosslinked synthetic network significantly stabilized gelatin against thermally induced gel-to-sol transitions at physiological temperatures. This prevented irreversible structural breakdown under heating–cooling cycles, preserving gel-like behavior. This stabilization addresses a key limitation of gelatin hydrogels and supports the use of these semi-IPN in environments exposed to fluctuating or elevated temperatures, such as *in vivo* implantation or thermally demanding processing steps.

Swelling, buoyancy, and degradation assays further indicate that the semi-IPN matrices possess moderate and tunable swelling capacity, extended floating times, and controllable degradation profiles that depend mainly on gelatin content. These properties align with requirements for oral delivery platforms, wound dressings, and resorbable scaffolds. The degradation behavior, accelerated at higher temperatures and higher gelatin loadings, suggests that matrix lifetime can be engineered by adjusting composition, providing design flexibility for short-term fillers, long-term structural supports, or staged-degradation constructs.

When considered collectively, the rheological, thermal, and degradation data identify systems in the G_3_-Xr_n_ and G_4_-Xr_n_ series as particularly promising candidates for tissue engineering, with formulations such as G_3_-Xr_2_, G_3_-Xr_3.5_, G_4_-Xr_2_, and G_4_-Xr_3.5_ being of particular interest. Their combination of high stiffness or strain tolerance, shear-thinning behavior, thermal robustness, and controlled degradability aligns closely with clinical demands for their use as load-bearing scaffolds, injectable cushioning gels, or 3D-printed bioinks for tissue-related applications. Further validation in model organisms is necessary to confirm their efficacy.

## Figures and Tables

**Table 1:** Preparation of gelatin-based IPN systems.

IPN Systems		Polymer 1										Polymer 2		Solvent	
		DF		*Furfuryl Group*	DM		*Maleimide Group*	TF ^†^			*Furfuryl Group*	Gelatin		H_2_O	DMSO
		mg	mmol	mmol	mg	mmol	mmol	mg	mmol	mol (%)	mmol	mg	% *w*/*v*	mL	mL
1	G_2_-Xr_2_	111	0.277	0.554	87	0.282	0.564	2	0.00387	2.0	0.0116	200	2	5	5
2	G_2_-Xr_3.5_	110	0.275	0.549	87	0.282	0.564	3	0.00580	3.5	0.0174	200	2	5	5
3	G_2_-Xr_5_	108	0.270	0.539	87	0.282	0.564	5	0.00967	5.0	0.0290	200	2	5	5
4	G_3_-Xr_2_	166	0.415	0.829	131	0.425	0.850	3	0.00580	2.0	0.0174	300	3	5	5
5	G_3_-Xr_3.5_	164	0.410	0.819	131	0.425	0.850	5	0.00967	3.5	0.0290	300	3	5	5
6	G_3_-Xr_3.5_	164	0.410	0.819	131	0.425	0.850	5	0.00967	3.5	0.0290	300	3	5	5
7	G_3_-Xr_5_	162	0.405	0.809	131	0.425	0.850	7	0.01354	5.0	0.0406	300	3	5	5
8	G_4_-Xr_2_	222	0.554	1.109	174	0.564	1.129	4	0.00774	2.0	0.0232	400	4	5	5
9	G_4_-Xr_3.5_	218	0.544	1.089	175	0.568	1.135	7	0.01354	3.5	0.0406	400	4	5	5
10	G_4_-Xr_5_	215	0.537	1.074	175	0.568	1.135	10	0.01934	5.0	0.0580	400	4	5	5

^†^ Sufficient quantity to react with 2.0, 3.5 or 5.0% of the maleimide groups from DM. DF: Disulfanediyl bis(ethane-2,1-diyl) bis[(furan-2-ylmethyl)carbamate]; DM: 1,8-dimaleimide-3,6-dioxaoctane; TF: 2,3,5-tri-*O*-[(furan-2-ylmethyl)carbamoyl]-d-ribono-1,4-lactone; and G*_m_*-Xr*_n_*: IPN with *m* gelatin and Polymer 1 concentration (% *w*/*v*) and *n* degree of crosslinking for Polymer 1 (mol %).

**Table 2:** Polymer concentration and targeted maleimide functional group percentage to react with furan rings of crosslinker (non-normalized and normalized data) used in the Box–Behnken design.

IPN System		Polymer Concentration (Gelatin and Synthetic Polymer) (Non-Normalized Values, % *w*/*v*)	Polymer Concentration (Gelatin and Synthetic Polymer) (Normalized Values)	Targeted mol (%) of Crosslinking ^†^ (Non-Normalized Values)	Targeted mol (%) of Crosslinking (Normalized Values)
1	G_2_-Xr_2_	2	−1	2	−1
2	G_2_-Xr_3.5_	2	−1	3.5	0
3	G_2_-Xr_5_	2	−1	5	+1
4	G_3_-Xr_2_	3	0	2	−1
5	G_3_-Xr_3.5_	3	0	3.5	0
6	G_3_-Xr_3.5_	3	0	3.5	0
7	G_3_-Xr_5_	3	0	5	+1
8	G_4_-Xr_2_	4	+1	2	−1
9	G_4_-Xr_3.5_	4	+1	3.5	0
10	G_4_-Xr_5_	4	+1	5	+1

^†^ Theoretical degree of crosslinking based on the percentage of global maleimide groups in Polymer 1 that can react with the furan rings from the crosslinker. G*_m_*-Xr*_n_*: IPN with *m* gelatin and Polymer 1 concentration (% *w*/*v*) and *n* degree of crosslinking for Polymer 1 (mol %).

**Table 3:** Selection of the bands of interest (wavenumber, $\bar{\nu }$, in cm^−1^) from the FTIR transmittance spectra of DF, DM, and DA polymer (POLDA).

Evolution in Intensity of the FTIR Bands During DA Polymerization ^†^	DF	DM	POLDA	Bands Suitable to Be Followed in Analysis of DA Polymerization by FTIR
Marked decrease	3309		3325	3309
**Almost undetectable**		**3098**		**3098**
No significant change in intensity	1681	1698	1690	
Small decrease	1538		1539	
Small decrease		1436	~1430	
Small decrease	1256		1243	
**Almost undetectable**	**820**	**832**		**820/832**
**Marked decrease**		**692**		**692**

^†^ The use of bold indicates relevant FTIR bands. DF: Disulfanediyl bis(ethane-2,1-diyl) bis[(furan-2-ylmethyl)carbamate]; DM: 1,8-dimaleimide-3,6-dioxaoctane; POLDA: resulting Diels–Alder polymer (Polymer 1).

**Table 4:** Rheological data from strain sweep and frequency sweep tests on the studied IPN.

IPN System		$\dot{\gamma_{c}}$ (%)	$G_{N}^{0}$ (Pa)	${G^{\prime }}_{1}$ (Pa)	${G^{\prime \prime }}_{1}$ (Pa)	$\eta_{0}$ (Pa·s)	$\eta_{1}^{*}$ (Pa·s)	tan (*δ*)
Blank G_2_		80.50	8.3	8.3	0.9	49.35	1.3	0.11
1	G_2_-Xr_2_	4.71	7583.2	7916.5	712.5	20,982.4	719.4	0.09
2	G_2_-Xr_3.5_	0.80	18.8	18.1	5.1	1386.0	15.4	0.29
3	G_2_-Xr_5_	4.02	41.3	41.5	4.7	267.99	6.6	0.11
Blank G_3_		40.31	30.6	30.6	3.7	184.06	4.9	0.12
4	G_3_-Xr_2_	0.75	14,493.0	14,100.4	3179.5	20,122.67	1295.1	0.23
5	G_3_-Xr_3.5_	0.40	1451.6	1417.4	428.0	4333.14	235.1	0.30
6	G_3_-Xr_3.5_	0.43	1430.9	1425.0	439.6	4320.58	249.7	0.31
7	G_3_-Xr_5_	2.03	631.9	551.4	123.5	1092.72	89.7	0.22
Blank G_4_		1.62	190.4	187.9	28.7	1187.37	30.3	0.15
8	G_4_-Xr_2_	6.26	5422.4	5028.1	928.1	10,210.41	811.8	0.18
9	G_4_-Xr_3.5_	12.79	472.6	476.3	64.9	2829.82	76.3	0.14
10	G_4_-Xr_5_	30.70	6.8	6.6	0.5	450.24	13.6	0.08

G*_m_*-Xr*_n_*: IPN with *m* gelatin and Polymer 1 concentration (% *w*/*v*) and *n* degree of crosslinking for Polymer 1 (mol %); Blank G*_m_*: gelatin colloidal solution at *m* concentration (% *w*/*v*). $\dot{\gamma_{c}}:$ Critical strain; $G_{N}^{0}$: plateau modulus; ${G^{\prime }}_{1}$: elastic modulus at 2π rad·s^−1^ (1 Hz); ${G^{\prime \prime }}_{1}$: viscous modulus at 2π rad·s^−1^ (1 Hz); $\eta_{0}$: zero-shear viscosity; $\eta_{1}^{*}$: complex viscosity at 2π rad·s^−1^ (1 Hz); and tan (δ) = ${G^{\prime \prime }}_{1}$/${G^{\prime }}_{1}$.

**Table 5:** Equations obtained for the studied rheological parameters as a function of the independent variables (normalized values) in the experimental design.

Equation	R^2^	df	F
${G^{\prime }}_{1}=-2837.58\cdot G^{2}+4241.15\cdot {Xr}^{2}-4412.56\cdot Xr+2252.98$	0.90	3.6	13.12
$\eta_{0}=6449.41\cdot {Xr}^{2}+3957.31\cdot G\cdot Xr-7438.26\cdot Xr+3217.5$	0.94	3.6	33.27
tan δ=−0.103·G2−0.113·Xr2−0.02·G·Xr+0.33	0.92	3.6	24.27
$\dot{\gamma_{c}}=9.123\cdot G^{2}+6.283\cdot G\cdot Xr+6.69\cdot G+4.27\cdot Xr+0.773$	0.92	4.5	14.51
$\eta_{1}^{*}=-286.82\cdot G^{2}+409.69\cdot {Xr}^{2}-436.05\cdot Xr+287.53$	0.97	3.6	54.99

$\dot{\gamma_{c}}$: Critical strain;$\;{G^{\prime }}_{1}$: elastic modulus at 2π rad·s^−1^ (1 Hz; Pa); $\eta_{0}$: zero-shear viscosity (Pa·s); $\eta_{1}^{*}$: complex viscosity at 2π rad·s^−1^ (1 Hz; Pa·s); tan (δ): ${G^{\prime \prime }}_{1}/{G^{\prime }}_{1}$; G: gelatin concentration (% *w*/*v*) in the IPN (normalized values); and Xr: crosslinking degree of Polymer 1 (normalized values).

**Table 6:** Variation in storage modulus and tan (δ) values with temperature during heating and cooling ramps (2 °C/min) for blank gelatin sample (4% *w*/*v*) and semi-IPN G_4_-Xr_3.5_. Values highlighted in purple are key temperature points identified in the thermal analysis.

**Stage**	**System**	**Para-meter**	**Temperature (°C)** **−** 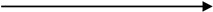 **+**										
			*25.00*	*32.14*	*32.55*	*37.91*	*38.15*	*40.37*	*44.92*	*59.90*	*63.50*	*64.03*	*65.01*
**Heating**	Blank G_4_	$G^{\prime }$ (Pa)	187.90	178.40	167.20	1.02	0.70	0.53	4.93	48.80	148.80	183.30	379.40
		tan (δ)	0.15	0.14	0.13	0.73	3.27	0.93	0.47	0.28	0.37	0.38	0.29
		*Trend*	gel-like, stable				sol-like			gel-like			
	G_4_-Xr_3.5_	$G^{\prime }$ (Pa)	475.31	453.59	454.38	323.61	313.64	277.16	243.24	245.59	245.18	258.16	277.00
		tan (δ)	0.14	0.22	0.18	0.21	0.21	0.17	0.24	0.19	0.31	0.25	0.17
		*Trend*	gel-like, stable			gel-like				gel-like, stable			
**Stage**	**System**	**Para-meter**	**Temperature (°C)** **−** 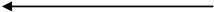 **+**										
			*25.00*	*32.14*	*32.55*	*37.91*	*38.15*	*40.37*	*44.92*	*59.90*	*63.50*	*64.03*	*65.01*
**Cooling**	Blank G_4_	$G^{\prime }$ (Pa)	0.14	0.10	0.11	0.16	0.15	0.21	0.14	32.20	178.60	277.00	361.77
		tan (δ)	1.08	1.56	0.88	0.71	0.78	0.74	0.73	0.23	0.28	0.28	0.29
		*Trend*	sol-like							gel-like			
	G_4_-Xr_3.5_	$G^{\prime }$ (Pa)	397.08	323.82	324.60	309.33	313.25	304.30	308.02	261.91	269.53	269.26	265.00
		tan (δ)	0.09	0.12	0.13	0.18	0.11	0.14	0.15	0.19	0.19	0.18	0.17
		*Trend*	gel-like, stable										

**Table 7:** Results of swelling, buoyancy, and degradability tests performed on the prepared IPN systems.

		Buoyancy Tests				Degradability Tests			
Name	Swelling Index (%)	FLT_1_ (min)	FT_1_ (min)	FLT_2_ (min)	FT_2_ (min)	*Stage 1* 25 °C/12 days	*Stage 2* 37 °C/48 h	*Stage 3* 37 °C/12 days	*Stage 4* 65 °C/48 h
G_2_-Xr_2_	135 ± 12	0	4	176	240	✗	✗	✗	**✓✓**
G_2_-Xr_5_	166 ± 35	0	4	66	>340	✗	✗	✗	**✓** ** ^§^**
G_3_-Xr_3.5_	264 ± 44	0	8	42	>360	✗	✗	**✓ ^†^**	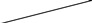
G_3_-Xr_5_	291 ± 41	0	60	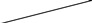	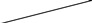	✗	✗	**✓ ^†^**	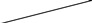
G_4_-Xr_2_	302 ± 58	0	9	51	360	✗	✗	**✓✓**	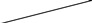
G_4_-Xr_3.5_	278 ± 22	0	40	60	120	✗	✗	**✓✓**	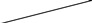
G_4_-Xr_5_	366 ± 5	0	9	111	>300	✗	✗	**✓✓**	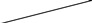

FLT_1_: Floating lag time 1; FT_1_: total floating time 1; FLT_2_: floating lag time 2; FT_2_: total floating time 2; G*_m_*-Xr*_n_*: IPN with *m* gelatin and Polymer 1 concentration (% *w*/*v*) and *n* degree of crosslinking for Polymer 1 (mol %). ✗: No degradation; **✓**: partial degradation with turbidity in the incubation medium; and **✓✓**: complete degradation. **^†^** Complete degradation in 19 days. **^§^** Complete degradation in 72 h.

**Table 8:** Main rheological features and potential biomedical applications for selected IPN systems.

IPN System		Rheological Remarks	Potential Biomedical Uses	Expected Performance
4	G_3_-Xr_2_	Excellent mechanical strength ($G_{N}^{0}$ ≈ 14 kPa; $\eta_{0}$ ≈ 20 kPa·s) tan (δ) = 0.23 → solid-like yet not brittle	Load-bearing scaffolds Bone/cartilage regeneration	+++
5/6	G_3_-Xr_3.5_	$G^{\prime }$ ≈ 1.4 kPa tan (δ) ≈ 0.30 → viscoelastic, deformable, suitable for extrusion	Injectable matrix Cartilage cushion 3D bioink for adipose/dermal tissue	+++
8	G_4_-Xr_2_	Strongest network [$G^{\prime }$ ≈ 5 kPa; $\eta_{0}$ ≈ 1–10 kPa·s; tan (δ) ≈ 0.18]	Injectable hydrogel Cartilage cushion	+++
9	G_4_-Xr_3.5_	Reduced $G^{\prime }$ (≈0.48 kPa) but higher $\dot{\gamma_{c}}$ Flexible	Bioink requiring flexibility and extrudability	++

G*_m_*-Xr*_n_*: IPN with *m* gelatin and Polymer 1 concentration (% *w*/*v*) and *n* degree of crosslinking for Polymer 1 (mol %); $\dot{\gamma_{c}}:$ critical strain;${\;G}_{N}^{0}$: plateau modulus; $G^{\prime }$: elastic modulus; $\eta_{0}$: zero-shear viscosity; and tan (δ) = $G^{\prime \prime }$/$G^{\prime }$. ++: Promising behavior; +++: very promising behavior.

## Data Availability

The original contributions presented in this study are included in the article. Further inquiries can be directed to the corresponding author.
